# Interdisciplinary vascular genetics evaluations in routine clinical care: insights from a five-year single-center experience

**DOI:** 10.1007/s00423-026-04090-7

**Published:** 2026-06-13

**Authors:** Sebastian Burkart, Sebastian Sailer, Daniel Körfer, Felix Marbach, Melanie Spanjaard, Steffen Hirsch, Heiko Brennenstuhl, Ilia Valentin, Katrin Hinderhofer, Julia Dennig, Lilian Kaufmann, Christian P. Schaaf, Dittmar Böckler, Nicola Dikow, Maja Hempel, Philipp Erhart

**Affiliations:** 1 Institute of Human Genetics, Heidelberg University Hospital, Heidelberg, Germany; 2 Institute of Human Genetics, Medical Faculty, University of Bonn, Bonn, Germany; 3 Department of Vascular and Endovascular Surgery, Heidelberg University Hospital, Heidelberg, Germany; 4 Institute of Human Genetics, Heidelberg University, Heidelberg, Germany

**Keywords:** Hereditary vascular disorders, Aortic aneurysm, Aortic dissection, Carotid Paraganglioma, Genetic testing, Multidisciplinary care, Precision medicine

## Abstract

**Purpose:**

Hereditary vascular disorders are clinically and genetically heterogeneous, predisposing patients to aneurysms, dissections, or vascular tumours. Despite advances in molecular diagnostics, integration of genetic testing into routine care remains an unmet need and a challenge of high clinical relevance.

**Methods:**

Implementation of an interdisciplinary outpatient clinic for vascular genetics evaluations. Patient selection based on current clinical recommendations for genetic diagnostics and counselling for vascular aneurysms/ dissections and carotid paraganglioma (CPGL). Single‑center, retrospective descriptive analysis of systematically recorded clinical and genetic data of all patients attending the Heidelberg specialized vascular-genetic outpatient clinic between 2021 and 2025.

**Results:**

We present five-year experience from a systematic and interdisciplinary clinical pathway for patients with aortopathies or carotid paraganglioma (CPGL), including pre‑visit data capture, interdisciplinary phenotyping, integrated pre‑ and post‑test counselling, and molecular guided clinical management. Pathological genetic findings in our aortic aneurysm/dissection and CPGL cohort were 16% (10/63) and 29% (10/34), respectively. The presence of multiple clinical predictors, defined as key risk indicators including early age at onset, multifocal disease, and positive family history, did not significantly influence the ratio of pathologic genetic findings in the aneurysm cohort, whereas a higher number of predictors was associated with increased number of pathogenic genetic variants in the CPGL cohort.

**Conclusion:**

A dedicated, interdisciplinary vascular genetics clinic can be feasibly implemented within routine clinical care. The proposed structured workflow provides a blueprint to systematically integrate genetics into vascular surgery and related clinical disciplines.

**Supplementary Information:**

The online version contains supplementary material available at 10.1007/s00423-026-04090-7.

## Introduction

Hereditary vascular disorders comprise a clinically and genetically heterogeneous group of conditions that predispose affected individuals to arterial aneurysms, dissections, or vascular tumors. These disorders are associated with a broad phenotypic spectrum, ranging from syndromic connective tissue diseases with systemic involvement to organ-specific vascular pathologies or tumor predisposition syndromes [1–5]. Despite their rarity at the individual level, hereditary vascular diseases collectively represent a clinically relevant entity due to their morbidity, mortality, more aggressive clinical course and implications for family members [6, 7].

The involved key biological pathways include extracellular matrix stability, transforming growth factor-β signaling, and others. Disruption of these pathways contributes directly to vessel wall fragility, abnormal vascular remodeling, or tumorigenesis [3, 4, 8, 9]. However, while molecular diagnostics have advanced rapidly, their translation into clinical care represents an unmet challenge.

Patients with suspected hereditary vascular disease are predominantly managed discipline-specific, most commonly by vascular surgery, cardiology, or interventional radiology. Usually, they are referred for genetic evaluation only in family cases or the occurrence of major complications [1, 10–13]. This fragmented care leads to delayed genetic assessment and can postpone conclusive diagnosis, prolong diagnostic uncertainty for patients and families, and limit implementation of gene-specific management strategies [13, 14]. This has direct consequences for clinical decision-making, including tailored surveillance intervals or individual surgical thresholds or risk-adapted operative procedures [1, 2, 4, 7].

The pronounced clinical heterogeneity of hereditary vascular disorders further underscores the need for specialized and interdisciplinary evaluation. Phenotypic features may be subtle and age-dependent, complicating risk stratification based on clinical criteria alone [1, 2, 4, 9]. A comprehensive assessment therefore requires the integration of detailed vascular phenotyping, systematic evaluation of extra-vascular features, and careful interpretation of family history. This is ideally performed by clinicians with complementary expertise [15]. Interdisciplinary collaboration between vascular surgeons and clinical geneticists is particularly critical, as it enables a shared assessment of vascular findings, surgical risk, and inherited disease features within a single, coordinated diagnostic setting [16, 17]. Published data of interdisciplinary clinical care remain limited and systematic evaluations of referral patterns, phenotypic characteristics, and diagnostic yield across a broad spectrum of hereditary vascular disorders are insufficient. Moreover, there is a lack of real-world data assessing the performance of integrated clinical care over extended time periods and across heterogeneous referral populations.

In this manuscript, we present the Heidelberg interdisciplinary vascular-genetic outpatient clinic by describing workflows and 97 individuals evaluated there between 2021 and 2025. We aim to provide a pragmatic and reproducible framework for integrating genetic diagnostics into vascular medicine. By systematically describing the structure, patient population, and outcomes of a specialized vascular-genetics clinic, we contribute to the evolving discussion on how precision medicine can be effectively implemented in the care of patients with hereditary vascular disease.

## Materials and methods

### Study population

This study was designed as a single-center, observational, descriptive analysis of a specialized outpatient clinic for vascular genetic disorders. The clinic provides interdisciplinary evaluation and genetic counseling for pre-selected individuals with suspected hereditary vascular disease. All consecutive patients evaluated between 2021 and 2025 were included. Preconsultation data collection and clinical consultations were systematically documented, and genetic testing results were consistently followed up. All patients gave their written consent for scientific utilization of their data.

### Data collection

Phenotyping was conducted by experienced clinicians including a clinical geneticist and a vascular surgeon. Vascular phenotypes were classified based on the primary reason for referral, the distribution of vascular involvement and comprehensive in-person phenotyping. All reported genetic variants were classified by the respective diagnostic laboratories according to ACMG criteria [18]. Genetic test results were grouped into the following reporting categories: (likely) pathogenic variant (LP/P), variant of unknown significance (VUS), no variant reported.

### Data curation

Data were retrospectively extracted from a structured, Excel-based clinical data set capturing routine outpatient care. The dataset comprised consecutive consultations and included the following variables: demographics (age at consultation, sex), referral characteristics (referral sources were categorized according to the referring medical specialty), clinical history (age at first symptom, reason for consultation), phenotypic features (vascular phenotype classified as multifocal versus isolated disease, clinical suspicion of an underlying connective tissue or syndromic disorder, presence of Marfan-like features with Systemic score, and Beighton hypermobility score), family history (a positive family history was defined as at least one affected first- or second-degree relative. When available, the number of affected family members was documented), genetic diagnostics (type of genetic test performed, genetic test result, reported genes/variants and ACMG variant classification, incidental or additional findings).

For downstream analysis the cohort was stratified using the presence of multiple clinical predictors for an underlying genetic cause, defined as key risk indicators including early age at onset (< 60 years), multifocal disease (> 2 different vascular regions), and positive family history (> 1 family member). Free-text clinical notes were retained for contextual interpretation but were not subjected to formal qualitative analysis.

### Statistical and computational analysis

Standard procedures of descriptive statistics were applied. Statistical comparisons between groups were performed in R using the non-parametric Wilcoxon rank-sum test (function wilcox.test), as implemented in the base *stats* package. Variables were illustrated using counts and percentages of the total cohort or on the total number of cases with available data on specific variables, unless stated otherwise. Missing data were not imputed. Data visualizations were generated in R using the *ggplot2* package, enabling consistent and reproducible graphical representation of the results [19].

AI assistance was limited to editorial functions and did not generate scientific claims. GPT5.2 was used to support language refinement, structural editing, and improvements in clarity and readability during manuscript preparation. All conceptual content, interpretation, and analytical conclusions were developed by the authors. All analyses and illustrations were performed using R (version 2025.09.2) environment for statistical computing and graphics (r-project.org).

## Results

### Establishment of clinical workflows to and within the specialized vascular genetics outpatient clinic

Patients are referred from a wide range of medical specialties to the vascular genetics outpatient clinic through a pre-defined, stepwise referral process. Request for an appointment could be done by clinicians or by patients themself contacting a defined service center. Prior to the scheduled visit, trained personnel systematically collect and review the patient’s medical records, personal health history, and three-generation family pedigree.

During the outpatient visit, a vascular surgeon and a clinical geneticist jointly perform a comprehensive phenotypic assessment. This interdisciplinary evaluation informs pre‑test genetic counseling, during which patients are assessed for eligibility for genetic testing, and the most appropriate testing strategy is selected (e.g., targeted gene panel, whole exome/genome sequencing). Genetic testing is offered based on pre-defined criteria (I) disease manifestation in thoracic aortic aneurysms and dissection under the age of 60 years [20], ; (II) positive familial history with vascular aneurysms or dissections; (III) multilocular appearance of arterial aneurysms or dissections (> 2 different vascular regions). In all patients with CPGL genetic testing was offered, irrespective of the presence of clinical indicators (IV). The choice of genetic testing modality was made on an individual clinical basis after interdisciplinary evaluation. Targeted gene panel testing was generally selected when the phenotype was consistent with a defined vascular disorder (non-syndromic or syndromic). Broader exome or genome sequencing was considered in patient with atypical, multisystemic presentations, or when the clinical symptoms were not sufficiently specific for one established diagnostic panel testing. Post‑test genetic counseling, again in an interdisciplinary setting, integrates molecular findings into individualized clinical care. This includes gene‑specific management recommendations, guidance on the selection and timing of surgical and/or interventional procedures, personalized surveillance protocols, and scheduled re‑evaluation. In addition, cascade testing of at‑risk family members is initiated to enable early diagnosis and preventive management.

**Fig. 1:**
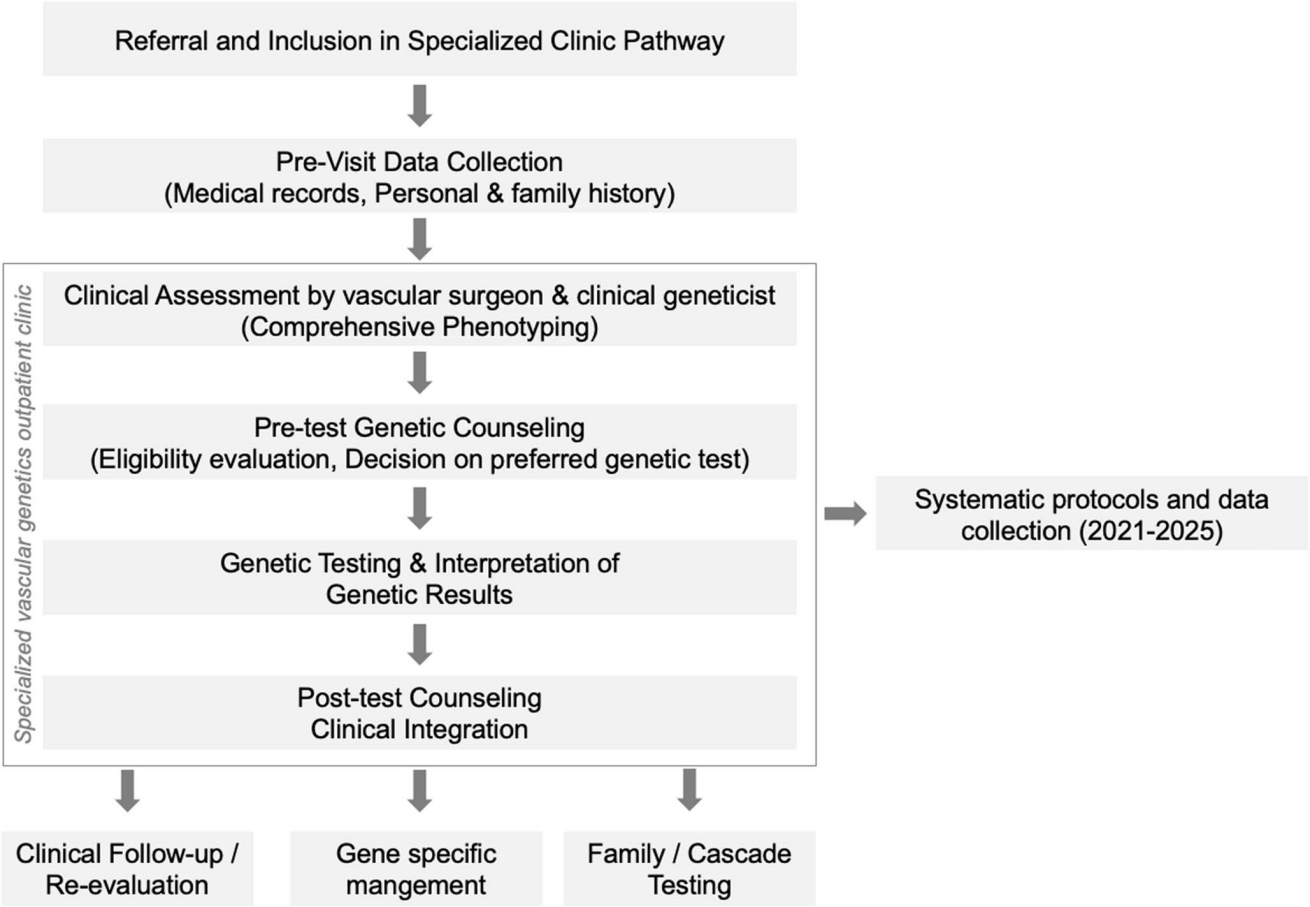
Patient flow in our specialized vascular genetics outpatient clinic. Following referral from various medical specialties, structured pre-visit clinical and family history data are collected and evaluated. During the visit comprehensive phenotyping by an experienced vascular surgeon and clinical geneticist is conducted. Eligible individuals undergo genetic counseling and testing, with results subsequently interpreted, communicated, and integrated into individualized and gene specific clinical management including clinical follow-ups and re-evaluation as well as testing of family members. Alongside the consultations in the specialized outpatient clinic, systematic protocols and data collection was conducted over a five-year period from 2021 to 2025

Throughout the entire workflow, systematic protocols with structured data collection are used to ensure that each patient receives consistent longitudinal care and enable the creation of a comprehensive clinical genetic record (Table 1). The overall workflow is illustrated in Fig. 1.

**Table 1:** Comprehensive clinical genetic record for individuals referred to the specialized vascular genetics outpatient clinic from January 2021 to December 2025 based on systematic protocols with structured data collection

ID	Age at consultation (y)	Sex	Reffering speciality	Age first symptom (y)	Symptom	Multifocal disease	Suspected Syndromic Disorder	Family history	Affected family members	Genetic test performed	Any reported variant	Genetic Result / Gene	Variant Classification (ACMG)	Incidental Finding	Genotype guided implication
Referal reason: Aneurysm / Dissection															
3	63	M	Vascular surgery	63	Typ-A aortic dissection	Yes	No	Yes	1	panel	No	no variant		No	
5	40	M	Center for Rare Diseases	36	Typ-A aortic dissection	No	No	No	0	genome sequencing	No	no variant		SH3TC2	Multidisciplinary care, cascade testing
6	53	M	Cardiology	NA	Family history	No	No	Yes	2	panel	No	no variant		No	
7	46	F	Primary Care	NA	Family history	No	No	Yes	2	family testing	NA	NA		No	
8	35	M	Vascular surgery	NA	Family history	No	No	Yes	3	family testing	NA	NA		No	
9	29	M	Vascular surgery	NA	Family history	No	No	Yes	3	family testing	NA	NA		No	
10	60	M	Vascular surgery	60	Typ-A aortic aneurysm	No	No	Yes	3	genome sequencing	No	no variant		No	
11	67	M	Vascular surgery	63	Typ-B aortic dissection	Yes	No	Yes	2	panel	Yes	TNXB	VUS	No	Close Re-Evaluation
12	53	M	Neurology	53	A. carotis dissection	Yes	No	No	0	panel	No	no variant		No	
15	64	M	Vascular surgery	35	Multiple Aneurysms	Yes	No	No	0	No testing performed	NA	NA		No	
16	48	M	Vascular surgery	NA	Family history	No	No	Yes	1	targeted testing	Yes	Deletion 10q23 including ACTA2	P	PTEN Deletion	Surgical intervention methods, Surveillance intervals, Cancer surveillance, cascade testing,
17	56	F	Vascular surgery	NA	Family history	No	No	Yes	5	exome sequencing	No	no variant		No	
18	66	M	Vascular surgery	52	Multiple Aneurysms	Yes	No	Yes	1	panel	No	no variant		No	
20	49	F	Clinical Genetics	49	Typ-A aortic dissection	No	No	Yes	1	exome sequencing	Yes	PRKG1	VUS	No	Close Re-Evaluation
21	19	M	Cardiothoracic Surgery	18	Typ-A aortic dissection	No	No	No	0	panel	Yes	TGFBR1	P	No	Surgical intervention methods and treshholds, Surveillance intervals, cascade testing,
22	66	M	Vascular surgery	64	Multiple Aneurysms	Yes	No	Yes	1	panel	No	no variant		Gonosomal mosaic (45,X0/46XY)	Furter investigation, family planing
23	53	M	Neurology	43	A. carotis dissection	Yes	No	No	0	panel	Yes	NOTCH1	VUS	No	Close Re-Evaluation
24	28	M	Vascular surgery	27	Typ-B aortic dissection	No	Yes	No	0	panel	Yes	FBN1	LP	No	Surgical intervention methods, Surveillance intervals, Ophthalmologic and Orthopaedic follow up, Cascade testing, Risk factor modification
28	42	F	Clinical Genetics	41	A. vertebralis dissection	Yes	Yes	No	0	panel	No	no variant		No	
36	26	F	Vascular surgery	26	Multiple Aneurysms	Yes	No	No	0	genome sequencing	Yes	TGFBR1	VUS	No	Close Re-Evaluation
37	33	F	Vascular surgery	33	Typ-A aortic dissection	Yes	No	No	0	genome sequencing	Yes	TGFBR2	LP	No	Surgical intervention methods and treshholds, Surveillance intervals, cascade testing,
38	45	M	Internal medicine	33	Typ-A aortic dissection	No	No	No	0	panel	No	no variant		No	
39	33	M	Neurology	32	A. vertebralis dissection	Yes	No	No	0	exome sequencing	No	no variant		No	
42	33	F	Vascular surgery	34	Family history	No	No	Yes	1	targeted testing	Yes	COL3A1	P	No	Surgical intervention methods and treshholds, Surveillance intervals, cascade testing,
44	40	M	Vascular surgery	40	Multiple Aneurysms	Yes	No	No	0	panel	Yes	COL3A1	VUS	No	Close Re-Evaluation
45	54	M	Vascular surgery	40	Multiple Aneurysms	Yes	Yes	Yes	1	panel	Yes	FBN1	P	No	
50	46	M	Vascular surgery	NA	Segregation	No	No	Yes	1	targeted testing	No	no variant		No	
51	51	M	Vascular surgery	NA	Segregation	No	No	Yes	1	targeted testing	No	no variant		No	
52	65	M	Vascular surgery	64	Multiple Aneurysms	Yes	No	Yes	2	panel	No	no variant		No	
55	56	M	Clinical Genetics	NA	Multiple Aneurysms	Yes	No	Yes	1	exome sequencing	Yes	ERG	VUS	No	Close Re-Evaluation
56	56	M	General surgery	32	A. vertebralis dissection	No	No	No	0	panel	No	no variant		No	
57	51	M	Vascular surgery	51	Dissection A. mesenterica superior	No	No	No	0	No testing performed	NA	NA		No	
58	40	M	Clinical Genetics	37	Typ-A aortic dissection	No	No	Yes	0	exome sequencing	Yes	MYLK	VUS	No	Close Re-Evaluation
59	57	M	Vascular surgery	57	Typ-A aortic dissection	Yes	No	Yes	1	panel	No	no variant		No	
60	27	F	NA	27	Multiple Aneurysms	Yes	No	Yes	2	panel	No	no variant		No	
62	65	M	Vascular surgery	NA	Multiple Aneurysms	Yes	No	Yes	2	panel	No	no variant		No	
63	63	M	Vascular surgery	NA	Typ-A aortic dissection	No	No	Yes	1	panel	Yes	ACTA2, FBN1	VUS	No	Close Re-Evaluation
65	62	F	Internal medicine	62	Typ-A aortic dissection	No	No	No	0	panel	No	no variant		No	
67	75	F	Clinical Genetics	75	Multiple Aneurysms	Yes	No	Yes	4	panel	No	no variant		No	
69	65	F	Vascular surgery	NA	Multiple Aneurysms	Yes	No	No	0	panel	Yes	COL3A1	VUS	No	Close Re-Evaluation
71	55	M	Vascular surgery	53	Typ-A aortic dissection	No	Yes	Yes	1	panel	Yes	FBN1	LP	No	Surgical intervention methods, Surveillance intervals, Ophthalmologic and Orthopaedic follow up, Cascade testing, Risk factor modification
73	65	M	Vascular surgery	65	Typ-A aortic aneurysm	No	No	Yes	1	panel	No	no variant		No	
74	65	M	Vascular surgery	65	Typ-A aortic dissection	No	No	Yes	1	panel	No	no variant		No	
75	63	M	Vascular surgery	63	Typ-A aortic dissection	Yes	Yes	Yes	1	panel	No	no variant		No	
76	56	M	Vascular surgery	54	Multiple Aneurysms	Yes	No	No	0	panel	No	no variant		No	
78	56	M	Vascular surgery	NA	Multiple Aneurysms	Yes	No	Yes	NA	panel	No	no variant		No	
79	41	F	Clinical Genetics	41	A. carotis dissection	Yes	Yes	No	0	exome sequencing	Yes	COL5A2	VUS	No	Close Re-Evaluation
80	38	F	Cardiothoracic Surgery	35	Typ-A aortic dissection	No	Yes	Yes	1	panel	No	no variant		No	
81	55	M	Primary Care	55	Multiple Aneurysms	Yes	No	Yes	4	panel	No	no variant		No	
82	64	M	Vascular surgery	NA	Multiple Aneurysms	Yes	No	Yes	2	panel	No	no variant		No	
84	62	M	Vascular surgery	NA	Typ-A aortic dissection	Yes	No	Yes	1	panel	No	no variant		No	
85	50	M	Clinical Genetics	50	Multiple Aneurysms	Yes	No	No	0	panel	No	no variant		No	
86	56	M	Primary Care	NA	Multiple Aneurysms	Yes	No	No	0	panel	Yes	NOTCH3	VUS	No	Close Re-Evaluation
87	60	F	Cardiology	NA	Typ-A aortic aneurysm	Yes	No	Yes	NA	panel	No	no variant		No	
88	44	M	Vascular surgery	38	Typ-B aortic dissection	No	No	Yes	1	panel	Yes	ACTA2	P	PTEN	Surgical intervention methods, Surveillance intervals, Cancer surveillance, cascade testing,
89	42	M	Vascular surgery	NA	Multiple Aneurysms	Yes	No	Yes	1	panel	No	no variant		No	
90	28	F	Clinical Genetics	NA	Multiple Aneurysms	Yes	No	Yes	1	panel	Yes	COL3A1	VUS	No	Close Re-Evaluation
91	50	M	Clinical Genetics	50	Multiple Aneurysms	Yes	No	Yes	1	panel	No	no variant		No	
92	82	F	Clinical Genetics	NA	Family history		No	Yes	2	No testing performed	NA	NA		No	
93	57	M	Clinical Genetics	56	Typ-A aortic aneurym	No	No	Yes	1	panel	Yes	FBN1	LP	No	Surgical intervention methods, Surveillance intervals, Ophthalmologic and Orthopaedic follow up, cascade testing, Risk factor modification
94	44	F	Vascular surgery	43	Typ-B aortic dissection	No	No	No	0	panel	Yes	FBN1	P	No	Surgical intervention methods, Surveillance intervals, Ophthalmologic and Orthopaedic follow up, cascade testing, Risk factor modification
95	46	F	Vascular surgery	46	Typ-B aortic dissection	No	No	No	0	panel	Yes	COL3A1	VUS	No	Close Re-Evaluation
97	49	F	Vascular surgery	49	Typ-A aortic dissection	No	No	No	0	panel	No	no variant		No	
Referal reason: Paraganglioma / Pheochromocytoma															
1	70	F	Vascular surgery	70	Paragangliom A. carotis	No	No	No	0	panel	No	no variant		No	
2	73	F	Vascular surgery	73	Paragangliom A. carotis	No	No	No	0	panel	No	no variant		No	
4	60	F	Vascular surgery	60	Paragangliom A. carotis	No	No	Yes	NA	panel	NA	NA		No	
13	38	M	Vascular surgery	38	Paragangliom A. carotis	No	No	No	0	panel	No	no variant		No	
14	37	M	Vascular surgery	36	Paragangliom A. carotis	No	No	No	0	panel	No	no variant		No	
19	42	F	Vascular surgery	36	Paragangliom A. carotis	No	No	Yes	1	targeted testing	Yes	SDHA	P	No	2–3 yearly follow up: Interdisciplinary tumour board; Endocrinological follow up; Cervical control MRI, Cascade testing
25	52	F	Vascular surgery	51	Paragangliom A. carotis	No	No	No	0	panel	No	no variant		No	
26	64	M	Vascular surgery	63	Paragangliom A. carotis	No	No	No	0	panel	No	no variant		No	
27	79	M	Vascular surgery	78	Paragangliom A. carotis	No	No	No	0	panel	No	no variant		No	
29	41	F	Vascular surgery	40	Paragangliom A. carotis	No	No	No	0	panel	No	no variant		No	
30	55	F	Vascular surgery	54	Paragangliom A. carotis	No	No	No	0	panel	No	no variant		No	
31	65	F	Vascular surgery	64	Paragangliom A. carotis	No	No	No	0	panel	No	no variant		No	
32	43	F	Vascular surgery	43	Paragangliom A. carotis	No	No	Yes	7	targeted testing	Yes	SDHD	P	No	2–3 yearly follow up: Interdisciplinary tumour board; Endocrinological follow up; Cervical control MRI, Cascade testing
33	42	F	Vascular surgery	NA	Family history	No	No	Yes	7	targeted testing	No	no variant		No	
34	35	F	Vascular surgery	30	Paragangliom A. carotis	No	No	Yes	7	targeted testing	Yes	SDHD	P	No	2–3 yearly follow up: Interdisciplinary tumour board; Endocrinological follow up; Cervical control MRI, Cascade testing
35	33	M	Vascular surgery	33	Paragangliom A. carotis	No	No	Yes	7	panel	Yes	SDHD	P	No	2–3 yearly follow up: Interdisciplinary tumour board; Endocrinological follow up; Cervical control MRI, Cascade testing
40	73	F	ENT	70	Paragangliom A. carotis	No	No	No	0	panel	No	no variant		No	
41	60	F	Vascular surgery	60	Multiple Aneurysms	Yes	No	No	0	panel	No	no variant		No	
43	68	M	Vascular surgery	68	Paragangliom A. carotis	Yes	No	No	0	panel	Yes	SDHD	P	No	2–3 yearly follow up: Interdisciplinary tumour board; Endocrinological follow up; Cervical control MRI, Cascade testing
46	42	F	Vascular surgery	36	Paragangliom A. carotis	No	No	Yes	1	panel	Yes	SDHA	P	No	2–3 yearly follow up: Interdisciplinary tumour board; Endocrinological follow up; Cervical control MRI, Cancer surveillance, cascade testing
47	48	F	Vascular surgery	44	Paragangliom A. carotis	No	No	Yes	2	panel	Yes	SDHB	P	CHEK2	2–3 yearly follow up: Interdisciplinary tumour board; Endocrinological follow up; Cervical control MRI, Cancer surveillance, cascade testing
48	53	M	Vascular surgery	26	Paragangliom A. carotis	Yes	No	Yes	1	panel	Yes	SDHD	P	MSH2	
49	65	F	ENT	55	Paragangliom A. carotis	Yes	No	No	0	panel	Yes	DLST	VUS	No	Close Re-Evaluation
53	72	F	ENT	70	Paragangliom A. carotis	Yes	No	No	0	panel	No	no variant		No	
54	66	M	Vascular surgery	66	Paragangliom A. carotis	No	No	Yes	1	Panel	No	no variant		No	
61	41	M	Vascular surgery	41	Paragangliom A. carotis	Yes	No	Yes	5	panel	Yes	SDHD	LP	No	2–3 yearly follow up: Interdisciplinary tumour board; Endocrinological follow up; Cervical control MRI, Cascade testing
64	71	F	ENT	68	Paragangliom A. carotis	No	No	Yes	1	panel	No	no variant		No	
66	65	F	ENT	63	Paragangliom A. carotis	No	No	Yes	NA	No testing performed	Yes	NA		No	
68	65	F	ENT	43	Paragangliom A. carotis	No	No	No	0	panel	No	no variant		No	
70	55	F	ENT	53	Paragangliom A. carotis	No	No	No	0	panel	No	no variant		No	
72	52	F	Vascular surgery	52	Paragangliom A. carotis	No	No	No	0	panel	Yes	SDHB	VUS	No	Close Re-Evaluation
77	60	F	Vascular surgery	60	Paragangliom A. carotis	No	No	Yes	NA	panel	Yes	SDHB	P	No	2–3 yearly follow up: Interdisciplinary tumour board; Endocrinological follow up; Cervical control MRI, Cascade testing
83	26	F	Vascular surgery	26	Paragangliom A. carotis	No	No	No	0	panel	Yes	SDHD	P	No	2–3 yearly follow up: Interdisciplinary tumour board; Endocrinological follow up; Cervical control MRI, Cascade testing
96	26	M	Vascular surgery	25	Paragangliom A. carotis	No	No	No	0	panel	No	no variant		No	

*NA* Not Available/Applicable, *F* Female, *M* Male

### Cohort characteristics and referral patterns

**Fig. 2:**
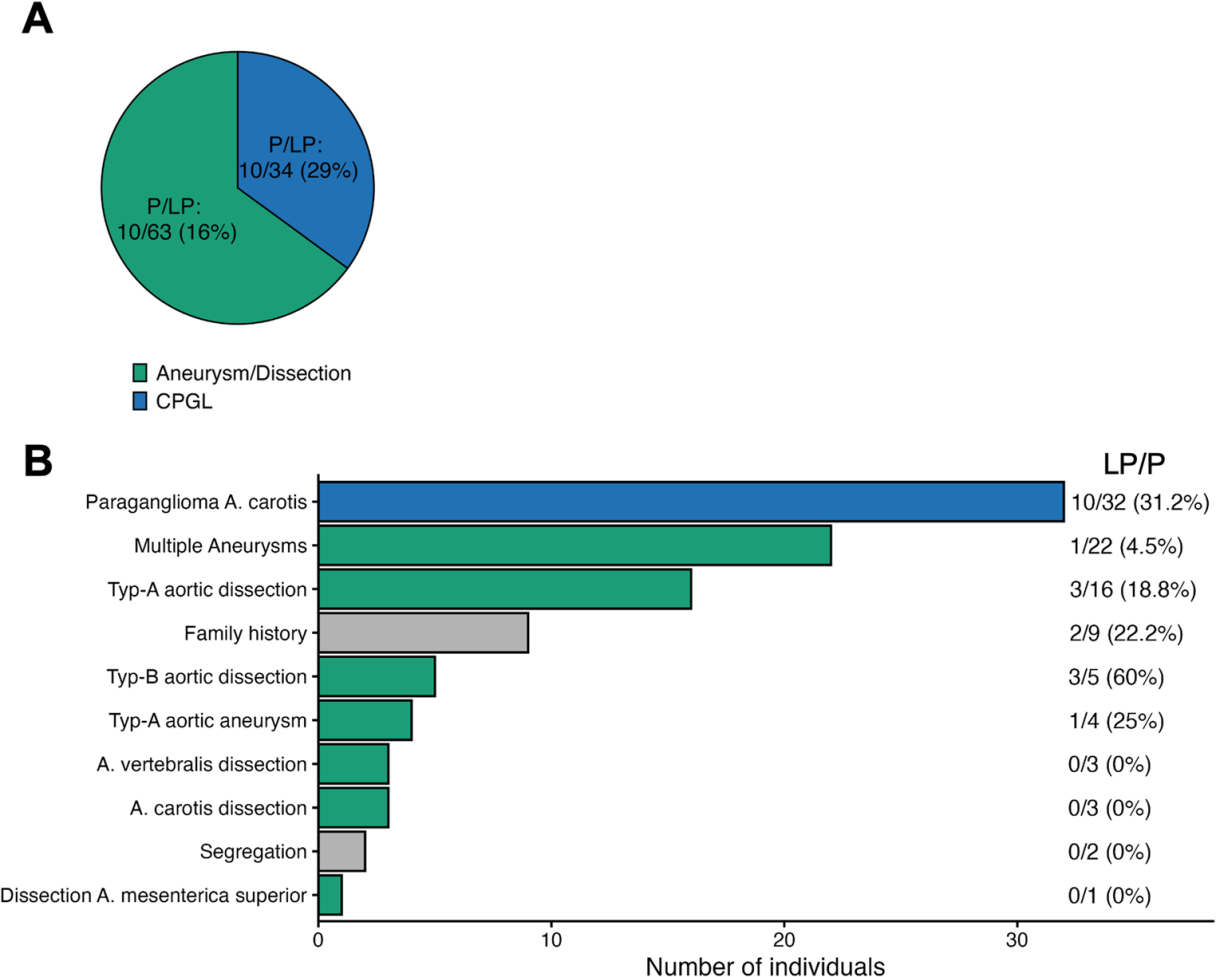
**A **Distribution of referral reasons among individuals. Pie chart illustrating the proportion of individuals referred to the specialized outpatient clinic for aortopathies/arteriopathies–related indications (green) and for CPGL–related indications (blue). Segment sizes correspond to the relative contribution of each referral reason to the total cohort. Diagnostic yield, as defined by the presence of pathogenic or likely pathogenic gene variants, is illustrated for each referral category. **B** Frequency of clinical symptoms at presentation. Horizontal bar chart summarizing the number of individuals presenting with each core symptom. Symptoms are ordered by frequency to facilitate comparison across categories. Bars are color-coded to indicate symptom grouping from A. Apparently healthy individuals with a positive family history or known familiar pathogenic variant for segregation are colored in grey. Diagnostic yield, as defined by the presence of pathogenic or likely pathogenic gene variants, is illustrated for each referral reason

We conducted a descriptive analysis of all patients referred to the specialized vascular genetics outpatient clinic from January 2021 to December 2025. The cohort comprised 97 individuals referred for a range of indications (Table 1, Fig. 2).

The most common reasons for referral were CPGL (*n* = 32, 33%), multiple aneurysms (*n* = 22, 23%), type‑A aortic dissection (*n* = 16,17%), and a positive family history of aneurysmal disease (*n* = 9, 9%) (Table 2). Overall, 63 patients (65% of the cohort) were affected by vascular aneurysms and/or dissections, whereas CPGL represented 34 patients (35%). Fortythree individuals were female (44%) and 54 were male (56%). Referral sources differed according to clinical indication: vascular surgery and genetics services served as the primary entry points for most patients (Supplementary Figure S1, Table 3).

**Table 2:** Distribution and frequency of different reasons for referral across the complete cohort

Reason for referral	Number of individuals
Paragangliom A. carotis	32 (33%)
Multiple Aneurysms	22 (22.7%)
Typ-A aortic dissection	16 (16.5%)
Family history	9 (9.3%)
Typ-B aortic dissection	5 (5.2%)
Typ-A aortic aneurysm	4 (4.1%)
A. carotis dissection	3 (3.1%)
A. vertebralis dissection	3 (3.1%)
Segregation	2 (2.1%)
Dissection A. mesenterica superior	1 (1%)

**Table 3:** Distribution and frequency of refering medical specialities across the complete cohort

Referral reason	Refering Medical Speciality	Number of individuals
Aneurysm	Vascular surgery	37 (58.7%)
	Clinical Genetics	11 (17.5%)
	Neurology	3 (4.8%)
	Primary Care	3 (4.8%)
	Cardiology	2 (3.2%)
	Cardiothoracic Surgery	2 (3.2%)
	Internal medicine	2 (3.2%)
	Center for Rare Diseases	1 (1.6%)
	General surgery	1 (1.6%)
	NA	1 (1.6%)
Paraganglioma	Vascular surgery	27 (79.4%)
	ENT	7 (20.6%)

### Patients referred for vascular aneurysm and/or dissection evaluation

We retrospectively screened the entire cohort to identify patients whose primary indication for consultation was related to aortopathies/arteriopathies. Sixty‑three individuals met this criterion during the study period and were included in the analysis.

The median age at consultation was 53 years (interquartile range [IQR] = 20 years), with a mean age of 51 (range 19–82 years). The median age at first symptom was 46 years. Gender distribution showed a predominance of males (70%, *n* = 44) over females (30%, *n* = 19). Multifocal aneurysmal disease, defined as involvement of ≥ 2 vascular territories, was documented in 52% (*n* = 33) of patients. At referral, clinical suspicion of an underlying syndromic disorder was recorded in 11% (*n* = 7). A positive family history (defined as at least one affected first‑ or second‑degree relative) was reported in 63% (*n* = 40). Among those with a positive family history, 20% (*n* = 8) had more than two affected relatives.

The majority of patients underwent targeted gene‑panel sequencing (68%, *n* = 43). Broad next‑generation sequencing approaches (whole exome or genome sequencing) were employed in 16% (*n* = 10). Four patients (6%, *n* = 4) received targeted testing for a known familial variant. In three cases, the initial recommendation was to test an affected family member first; no genetic testing was performed in three additional cases due patient preferences.

Genetic results were categorized as follows: (likely) pathogenic or pathogenic variants in 16% (*n* = 10), variants of uncertain significance in 21% (*n* = 13), and no clinically relevant variant identified in 63% (*n* = 40). Incidental findings were reported in four patients, comprising two germline *PTEN* and one *SH3TC2* variant as well as signs of a gonosomal mosaic (45,X0/46,XY).

Within the group of patients with aortopathies/arteriopathies the absolute amount of clinical predictor was as follows: positive for one indicator in ~ 37% (*n* = 23), positive for two indicators in ~ 50% (*n* = 32), positive for three indicators in ~ 10% (*n* = 6), and none in ~ 3% (*n* = 2) (Supplementary table S2). Interestingly, Kruskal–Wallis rank-sum test did not reveal a statistically significant difference (*p* = 0.983) regarding the presence of (likely) pathogenic variants between clinical predictor counts for the complete aortopathies/arteriopathies cohort. In the next step, we analyzed the predictive value of clinical predictors in two subgroups with multiple aneurysms (*n* = 21) or thoracic aortic aneurysms [20]. Again, Kruskal–Wallis rank-sum test did not reveal statistically significant differences in both subgroups (*p* = 0.112 and *p* = 0.767, respectively).

We additionally analyzed whether clinical suspicion of a syndromic connective tissue disorder was associated with increased diagnostic yield. Individuals with clinically suspected syndromic disease showed higher odds of harboring a pathogenic or likely pathogenic variant (OR 5.05, 95% CI 0.61–37.63), although this did not reach formal statistical significance (Fisher’s exact test, *p* = 0.073). Additional exploratory analyses evaluating younger age-at-onset thresholds (e.g. <30 years) did not demonstrate a statistically significant association between younger age at first symptom onset and the presence of LP/P variants. Table 1 summarizes some of the gene specific consequences and implications for clinical management. In all cases with pathogenic variants, the genetic results directly guided surveillance plans, preventive strategies, and individualized management, and allowed targeted testing of at-risk family members.

### Patients referred for carotid paraganglioma evaluation

During the study period, 34 patients were referred for comprehensive evaluation of carotid paraganglioma (CPGL). The majority (80%) were referred by vascular surgeons, while 20% originated from otolaryngology, reflecting cases with vascular invasion and necessity for surgical cooperation.

The median age at time of initial consultation was 55 years (inter‑quartile range [IQR] = 22 years), and the mean age was 54 (range: 26–79 years). The median age at first symptom was 51 years. Females constituted 71% (*n* = 24) of the cohort, whereas males comprised 29% (*n* = 10). Multifocal disease was identified in 6 patients (18%). No individual showed clinical criteria for a complex, syndromic disorder. A positive family history of paraganglioma or pheochromocytoma was reported in 41% (*n* = 14) of patients; among these, 57% (*n* = 8) had more than two affected relatives.

Gene‑panel sequencing covering the most frequently altered genes in paraganglioma and pheochromocytoma was performed in 85% (*n* = 29) of the cohort. Four patients (12%) received targeted testing for a known familial variant identified in their relatives. One patient was not tested due to patient preference. Pathogenic or likely pathogenic variants were detected in 10 patients (29%). Variants of uncertain significance (VUS) were identified in two patients (6%), and 22 patients (65%) had no pathogenic or VUS detected. Incidental germline findings of clinical significance were reported in two patients: a *CHEK2* and an *MSH2* pathogenic variant. Table 1 summarizes some of the gene specific implications for clinical management.

Even genetical diagnostics was conducted in all CPGL patients, we performed analysis of the predictive value of the presence of one or more major clinical predictors. 18 patients (53%) had one indicator, 7 patients (20%) had two indicators, and 2 patients (6%) had three indicators. 7 patients (21%) lacked any of these risk factors. Kruskal–Wallis rank-sum test did reveal a statistically significant difference (*p* < 0,001) regarding the presence of (likely) pathogenic variants between clinical predictor counts, with an enrichment for clinical indicators in the group with reported LP/P variants. Individuals with LP/P variants often were positive for 2 or more clinical predictors in their medical history (Supplementary Figure S3).

## Discussion

This manuscript provides an overview of the structure and experience of a specialized vascular genetics outpatient clinic over a five-year period. By integrating referral patterns, phenotypic presentations, and genetic testing outcomes, it illustrates how coordinated clinical and genetic expertise can be implemented in routine care for individuals with suspected hereditary vascular disorders.

The described cohort spans a wide phenotypic spectrum, ranging from isolated vascular lesions to complex, multifocal disease including aneurysms and dissections as well as vascular tumors. This heterogeneity highlights the importance of well-defined referral criteria and sustained interdisciplinary collaboration to ensure appropriate patient selection and comprehensive evaluation [14]. Notably, a substantial proportion of individuals were referred based on manifest vascular disease rather than a known family history. This finding underscores the clinical value of phenotype-driven genetic assessment, particularly in settings where hereditary risk may not be immediately apparent.

In patients referred for (multiple) aneurysm or dissection, common clinical indicators such as family history, multifocal disease, early disease onset or the combination of several clinical predictors showed similar diagnostic yields. This suggests that genetic testing should be considered even in the absence of a strong a priori suspicion of a hereditary vascular disorder [1–3, 7, 10–12, 21]. Traditional clinical predictors alone are not sufficient to reliably identify monogenic disease in this setting and support a broader, phenotype-driven testing approach [11, 12, 21]. While younger age at symptom onset was not significantly associated with LP/P variants, clinically suspected syndromic disease was associated with a trend toward higher diagnostic yield, emphasizing the importance of comprehensive phenotypic assessment. Overall, the diagnostic yield in this group was within the expected range, with pathogenic or likely pathogenic variants detected in 16% of individuals, variants of uncertain significance (VUS) in 21%, and negative results in 63% [22–26].

In contrast, in the CPGL cohort, the presence and combination of multiple clinical predictors were associated with a higher diagnostic yield. This finding shows that risk-based testing strategies remain effective in CPGL, which is consistent with the well-established genotype–phenotype relationships for this disease [4, 5, 27]. From a practice-oriented perspective, the applied genetic testing for all CPGL have still proven robust, feasible, and clinically effective, particularly with respect to the observed diagnostic yield in carotid paraganglioma, compatible with literature estimates [28–30].

We established a structured, stepwise clinical workup (Fig. 1) for: [1] systematic pre-visit collection of clinical and family history data; [2] joint evaluation by an experienced vascular surgeon and a clinical geneticist; [3] integrated phenotypic assessment with coordinated diagnostic and management decisions and [4] provision of both pre- and post-test genetic counseling within the same clinical setting. A further strength of this model is the joint phenotyping performed by experienced vascular surgeons and clinical geneticists. The interdisciplinary evaluation enhances diagnostic accuracy and improves clinical interpretation of the comprehensive phenotypic assessment. Although interdisciplinary consultations require additional organizational effort, this model reduces redundant appointments, shortens diagnostic timelines, and enables real-time, consensus-based decision-making. Importantly, the close integration of clinical evaluation and genetic counseling facilitates the direct translation of molecular findings into individualized, gene-specific management recommendations [1, 3–5, 27, 31]. Identifying a genetic diagnosis had clear and immediate benefits for patient care (Table 1) [1, 11, 12]. This includes cascade testing for early identification and risk stratification in affected families; genotype-adapted follow-up and surveillance strategies and gene-guide therapy, particularly in aortopathies and arteriopathies, where genotype-specific risks support timely intervention and often favor open surgical approaches [5].

By bringing vascular medicine and genetics together in one clinical setting, the specialized vascular genetics outpatient clinic fills an important gap in routine care. Collecting clinical and genetic data as part of everyday practice increases the real-world relevance of the findings and supports their broader applicability. The standardized clinic structure also makes this model reproducible and suitable for implementation at other centers. Collectively, these findings highlight the role of dedicated vascular genetics clinics as effective platforms for implementing precision medicine in vascular disease.

### Limitations & Future perspectives

This study has limitations and faces methodological challenges, which are important to consider for interpretation. First, it is a single-center, retrospective analysis with a moderate sample size, which limits generalizability and does not allow robust conclusions on long-term clinical outcomes. Second, referral bias is likely, as individuals with more severe or complex phenotypes are more frequently referred to a specialized clinic; for example in the CPGL group we have an enrichment for cases with vascular invasion. This reflects real-world referral pathways but may influence diagnostic yield estimates. Third, genetic testing strategies were phenotype-driven and therefore not fully uniform across all individuals. While this mirrors routine clinical practice, it limits direct comparison of diagnostic yields between subgroups.

Future prospective, multicenter studies are needed to validate referral criteria, clinical predictors, and diagnostic algorithms across different healthcare settings. Integrating the vascular genetics outpatient clinic into a prospective registry with predefined clinical endpoints would allow outcome-focused analyses (e.g. for dissection incidence, timing and outcome of surgical intervention). Longitudinal follow-up will be essential to refine genotype–phenotype correlations and to assess how genetic diagnoses influence clinical management and prognosis over time [3]. Engagement with patient advocacy groups could support long-term follow-up, improve study participation, and strengthen patient-centered outcome measures.

Distinct from the vascular genetics outpatient clinic, one important goal is to enable genotype-driven medical care even during acute vascular events, such as aortic dissection. To achieve this, genetic testing workflows must be fast enough to inform clinical decision-making before or alongside surgical and interventional treatment. As many vascular emergencies require direct action, this concept refers to subacute cases where genetic testing should be initiated at first presentation and integrated into specialized ultra-rapid diagnostic pathways to ensure timely and clinically meaningful results.

## Conclusion

Overall, this study demonstrates that a dedicated vascular genetics outpatient clinic delivers diagnostic and clinical value. By integrating interdisciplinary phenotyping, genetic testing, and counseling within a standardized care workup, the model facilitates accurate diagnosis, informed management, and longitudinal patient care. The presented approach may serve as a practical blueprint for similar initiatives aiming to systematically integrate genetics into vascular surgery and related clinical disciplines.

## Electronic Supplementary Material

Below is the link to the electronic supplementary material.

Supplementary figure 1 (PNG 432 KB)

High Resolution Image (TIFF 18.2 MB)

Supplementary figure 2 (PNG 418 KB)

High Resolution Image (TIFF 18.2 MB)

Supplementary figure 3 (PNG 432 KB)

High Resolution Image (TIFF 410 KB)

## Data Availability

No datasets were generated or analysed during the current study.
