# Supplementary figures and images for: Interdisciplinary vascular genetics evaluations in routine clinical care: insights from a five-year single-center experience
Source: Langenbecks Arch Surg. 2026 Jun 13;411(1):162. doi: 10.1007/s00423-026-04090-7 (PMC13264565; doi:10.1007/s00423-026-04090-7)

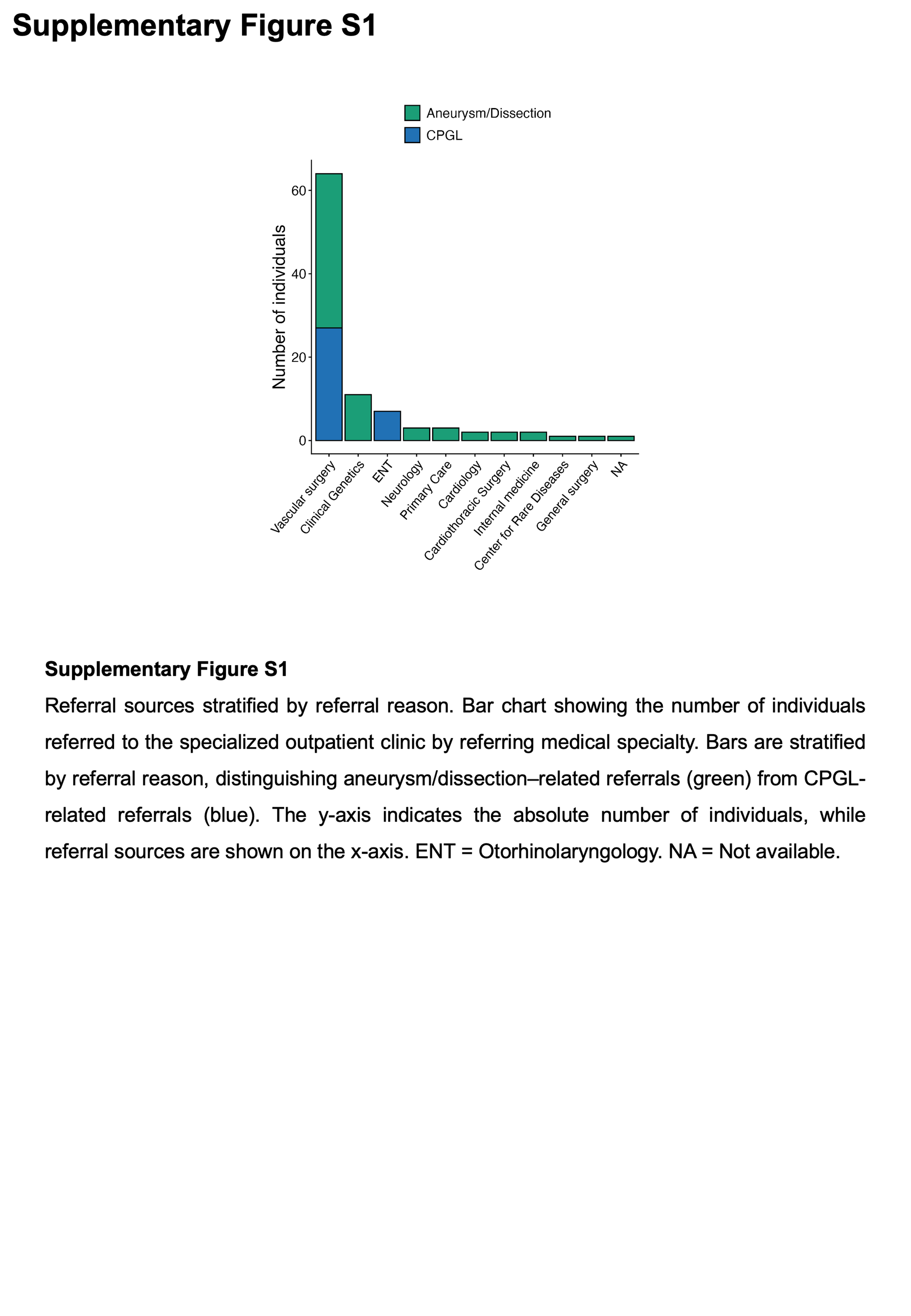

Supplement: Supplementary file 1 — (PNG 432 KB) [file 423_2026_4090_Fig3_ESM.png]

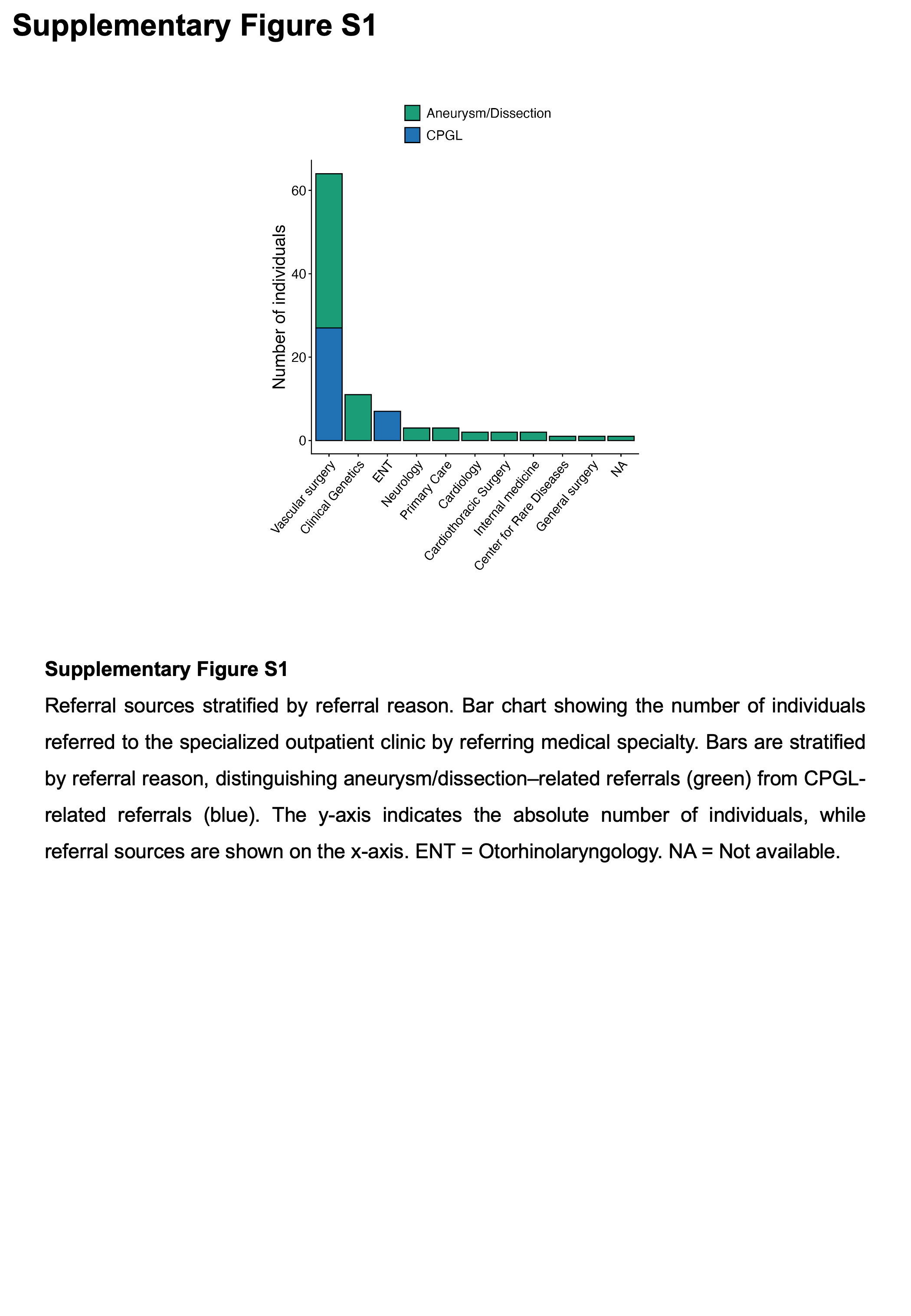

Supplement: Supplementary file 2 — High Resolution Image (TIFF 18.2 MB) [file 423_2026_4090_MOESM1_ESM.tiff]

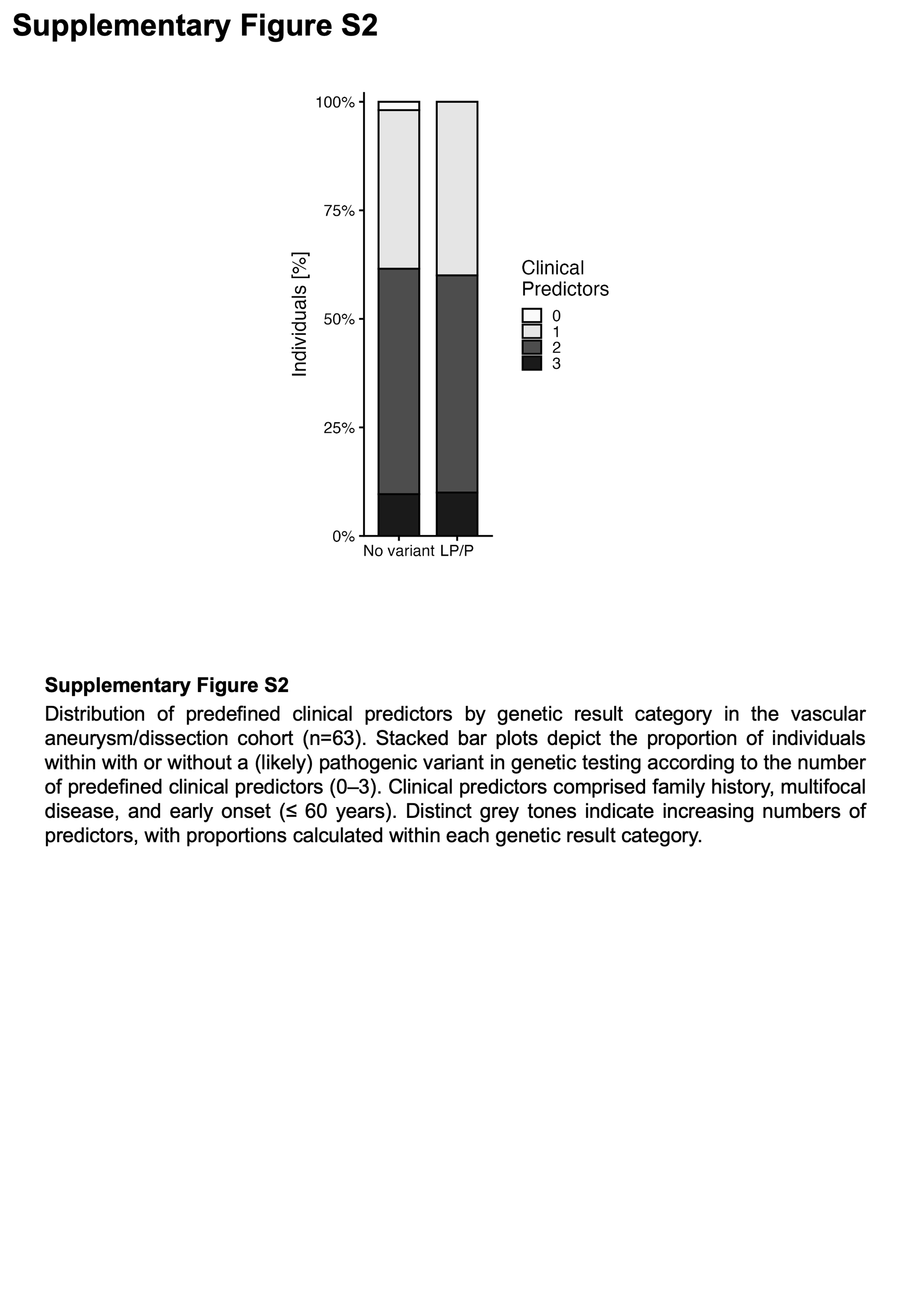

Supplement: Supplementary file 3 — (PNG 418 KB) [file 423_2026_4090_Fig4_ESM.png]

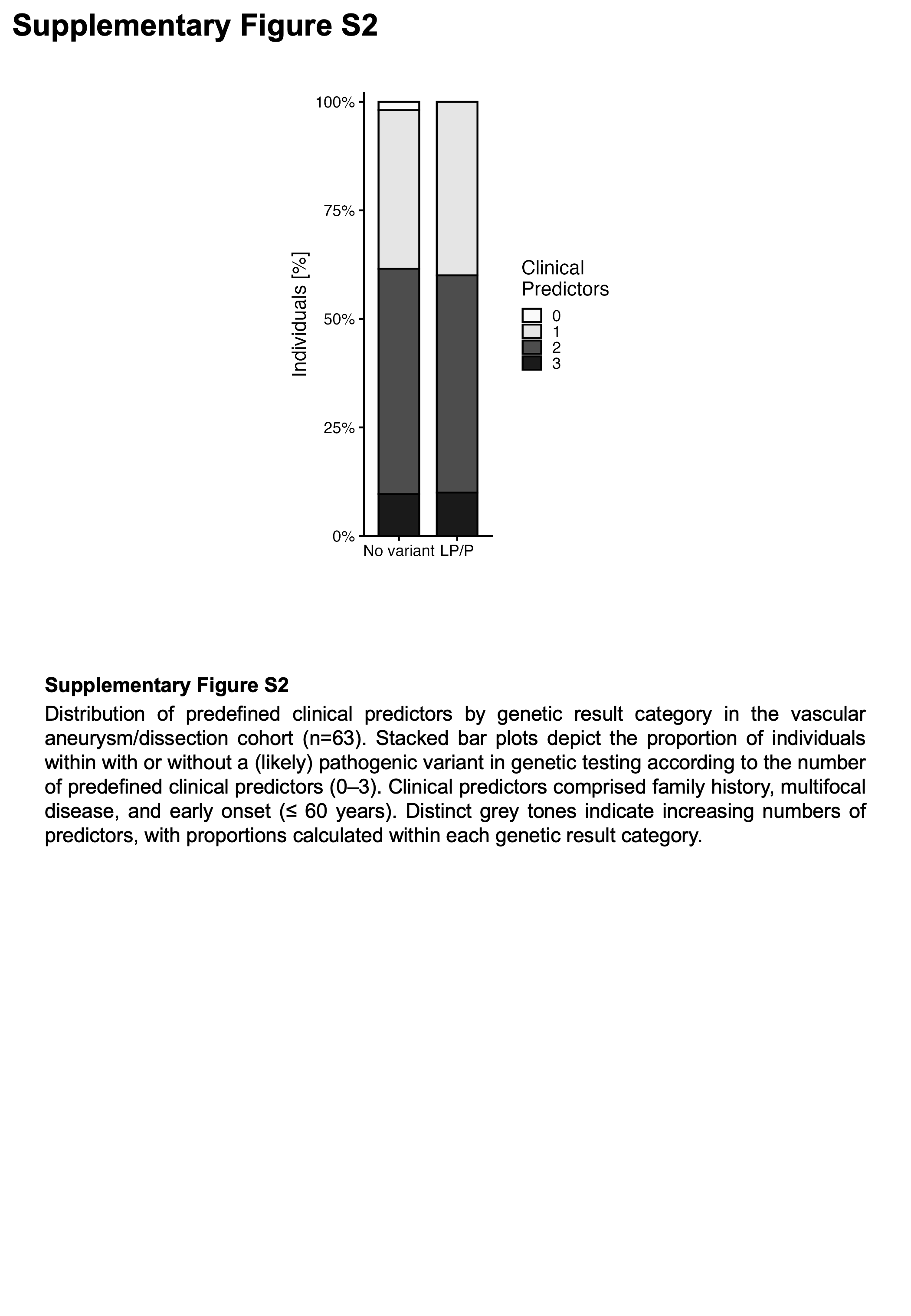

Supplement: Supplementary file 4 — High Resolution Image (TIFF 18.2 MB) [file 423_2026_4090_MOESM2_ESM.tiff]

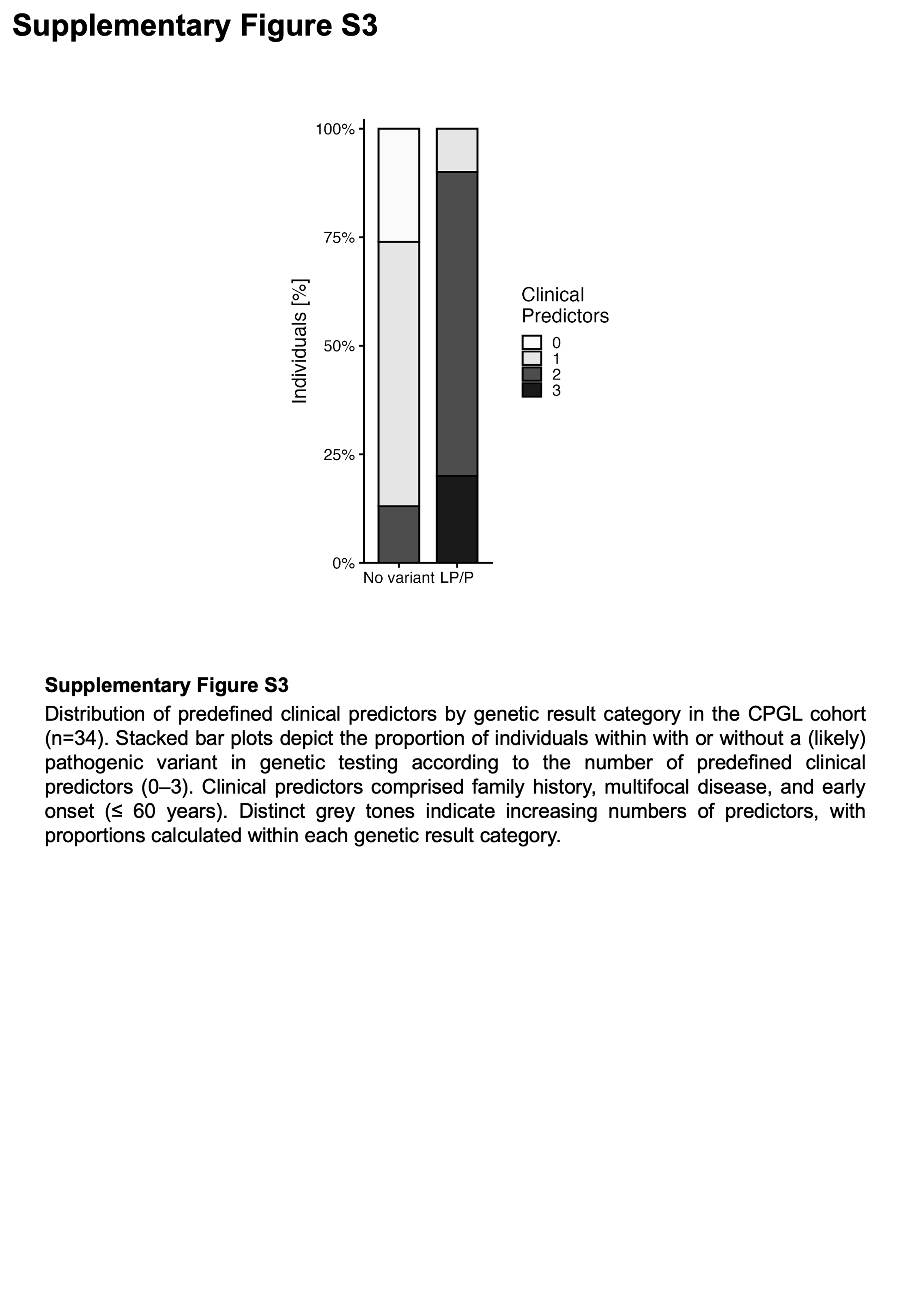

Supplement: Supplementary file 5 — (PNG 432 KB) [file 423_2026_4090_Fig5_ESM.png]

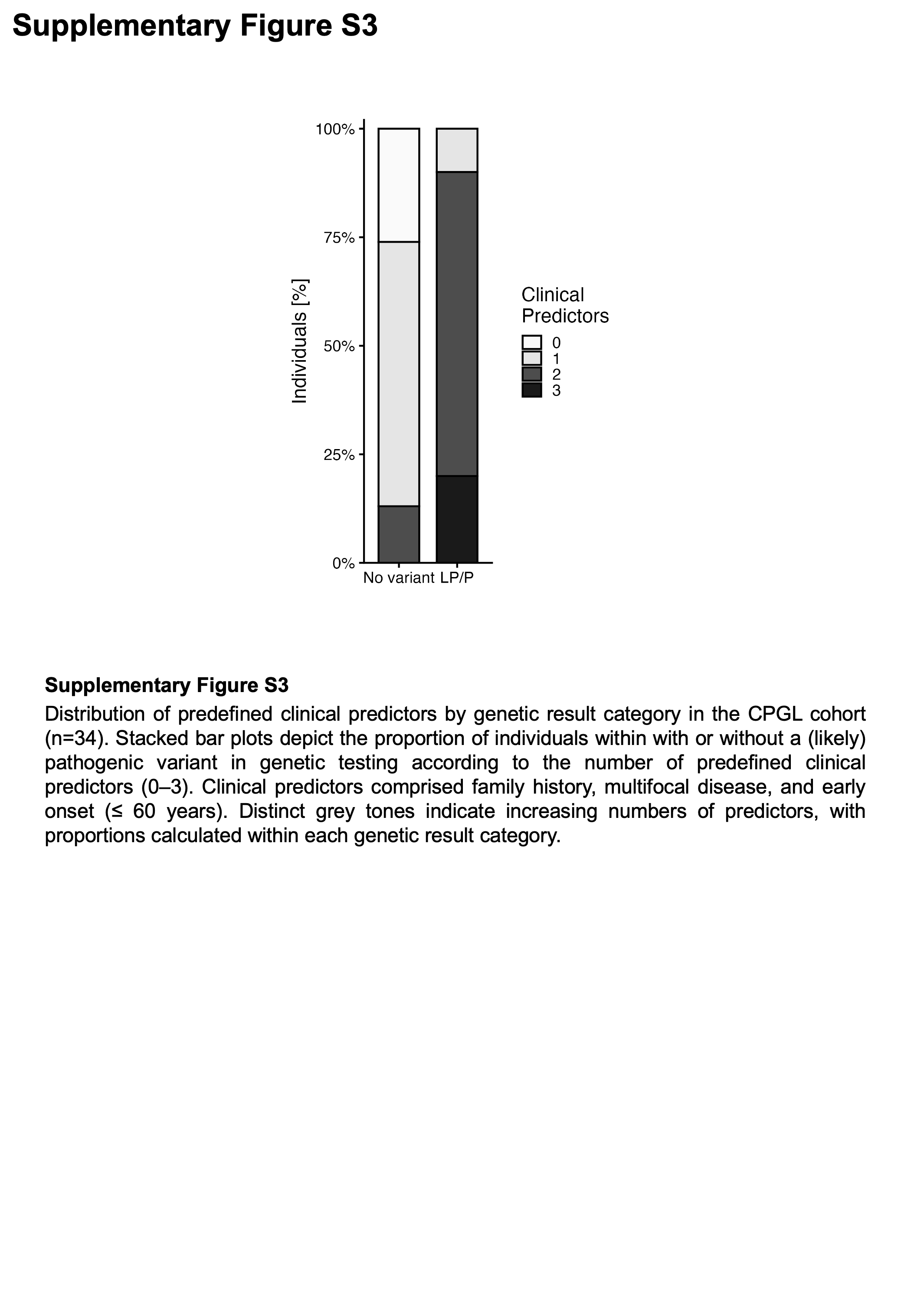

Supplement: Supplementary file 6 — High Resolution Image (TIFF 410 KB) [file 423_2026_4090_MOESM3_ESM.tiff]
